# Demographic and Behavioral Disparities in Skin Cancer Among HS Patients

**DOI:** 10.51894/001c.143949

**Published:** 2025-09-30

**Authors:** Jimmy Le

**Affiliations:** 1 College of Osteopathic Medicine Michigan State University

## 28

### BACKGROUND

Hidradenitis suppurativa (HS) is a chronic inflammatory skin disease associated with risk of cutaneous squamous cell carcinoma (cSCC), basal cell carcinoma (BCC) and melanoma. This study examines demographic disparities and behavioral risk factors for skin cancer development in HS using a longitudinal dataset of 13,130 patients from Henry Ford (1995–2022).

### METHODS

Retrospective analysis of demographics (race, sex, age) and behavioral risk factors (tobacco, alcohol use) was performed. Chi-square tests were used to compare HS patients with and without cancer.

### RESULTS

Among 13,130 HS cases, 0.63% (n = 83) had BCC, 0.60% (n = 79) had melanoma, and 0.49% (n = 64) had SCC. White patients (n = 4,772, 36.34%) had the highest incidence of BCC (89.2%, 74/83), SCC (71.9%, 46/64), and melanoma (64.6%, 51/79), whereas Black patients (n = 7,109, 54.14%) had lower incidences of BCC (4.8%, 4/83) and SCC (18.8%, 12/64), but a higher melanoma frequency (31.65%, 25/79) compared to Whites. Men (n = 9,652, 73.5%) were more frequently diagnosed with BCC (56.63%, n = 47) and SCC (54.69%, n = 35), whereas melanoma was more common in women (78.48%, n = 62). The mean age at first diagnosis was younger in females for BCC (52.6 vs. 60.6 years), melanoma (45.6 vs. 53.2 years), and SCC (54.5 vs. 64.9 years) compared to males, respectively. Tobacco use was significantly associated with overall cancer incidence (p ≤ 0.001) but not with specific cancer types. Alcohol use trended significantly overall (p = 0.0475) but was not associated with BCC (p = 0.1210), SCC (p = 0.1131), or melanoma (p = 0.8548).

### CONCLUSION

Skin cancer incidence among HS patients was influenced by race, gender, and behavioral disparities. The prevalence of BCC, SCC, and melanoma in White patients was disproportionately high, compared to Black patients. Men were more frequently diagnosed with BCC and SCC, while melanoma was more common in women. Both Tobacco and alcohol use were significantly associated with cancer risk in HS. These findings highlight the urgent need for more precise risk factor assessments, improved demographic data collection, and targeted prevention strategies to enhance early detection and intervention for high-risk populations.

